# User-Centered Design and Usability of Voxe as a Pediatric Electronic Patient-Reported Outcome Measure Platform: Mixed Methods Evaluation Study

**DOI:** 10.2196/57984

**Published:** 2024-09-19

**Authors:** Samantha J Anthony, Sarah J Pol, Enid K Selkirk, Amarens Matthiesen, Robert J Klaassen, Dorin Manase, Amanda Silva, Melanie Barwick, Jennifer N Stinson, Alameen Damer, Mowa Ayibiowu, Selina X Dong, Stephan Oreskovich, Michael Brudno

**Affiliations:** 1 Child Health Evaluative Sciences Research Institute, Peter Gilgan Centre for Research and Learning The Hospital for Sick Children Toronto, ON Canada; 2 Transplant and Regenerative Medicine Centre The Hospital for Sick Children Toronto, ON Canada; 3 Factor-Inwentash Faculty of Social Work University of Toronto Toronto, ON Canada; 4 Comprehensive Hemophilia Care Clinic Children's Hospital of Eastern Ontario Ottawa, ON Canada; 5 Department of Pediatrics University of Ottawa Ottawa, ON Canada; 6 The Data Aggregation, Translation and Architecture (DATA) Team University Health Network Toronto, ON Canada; 7 Dalla Lana School of Public Health University of Toronto Toronto, ON Canada; 8 Department of Psychiatry University of Toronto Toronto, ON Canada; 9 Lawrence S Bloomberg Faculty of Nursing University of Toronto Toronto, ON Canada; 10 Department of Computer Science University of Toronto Toronto, ON Canada

**Keywords:** eHealth, end user engagement, mobile phone, patient-reported outcome, patient-reported outcome measures, pediatric, user-centered design

## Abstract

**Background:**

Electronic patient-reported outcome measures (ePROMs) are standardized digital instruments integrated into clinical care to collect subjective data regarding patients’ health-related quality of life, functional status, and symptoms. In documenting patient-reported progress, ePROMs can guide treatment decisions and encourage measurement-based care practices. Voxe is a pediatric and user-centered ePROM platform for patients with chronic health conditions.

**Objective:**

We aimed to describe the user-centered design approach involving feedback from end users and usability testing of Voxe’s platform features to support implementation in a pediatric health care setting.

**Methods:**

Purposive sampling was used to recruit patients aged 8-17 years from 2 chronic illness populations in 2 pediatric hospitals in Canada. Patients’ health care team members were also purposively recruited. One-on-one iterative testing sessions were conducted digitally by research team members with participants to obtain feedback on the appearance and functionalities of the Voxe platform prototype. Patients and health care providers (HCPs) completed Voxe-related task-based activities. International Organization for Standardization key performance indicators were tracked during HCP task-based activities. HCPs also completed the System Usability Scale. To test platform usability, the think-aloud technique was used by participants during the completion of structured tasks. After completing all task-based activities, patient participants selected 5 words from the Microsoft Desirability Toolkit to describe their overall impression and experience with the Voxe platform. Qualitative data about likes, dislikes, and ease of use were collected through semistructured interviews. Feedback testing sessions were conducted with patients and HCPs until Voxe was acceptable to participating end users, with no further refinements identified. Quantitative and qualitative data analysis were completed using descriptive statistics and content analysis.

**Results:**

A total of 49 patients and 38 HCPs were recruited. Patients were positive about Voxe’s child-centered design characteristics and notification settings. HCPs rated Voxe as user-friendly and efficient, with the time to complete tasks decreasing over time. HCPs were satisfied with the Voxe platform functionalities and identified the value of Voxe’s system notifications, summarized display of ePROM results, and its capacity to integrate with electronic medical records. Patients’ and HCPs’ high satisfaction rates with the Voxe prototype highlight the importance of being responsive to user suggestions from the inception of eHealth platform developments to ensure their efficient and effective design.

**Conclusions:**

This paper describes the user-centered creation and usability testing of Voxe as an ePROM platform for implementation into clinical care for pediatric patients with chronic health conditions. As a patient-facing platform that can be integrated into electronic medical records, Voxe aligns with measurement-based care practices to foster quality patient-centered approaches to care. End users’ positive feedback and evaluation of the platform’s user-friendliness and efficiency suggest that Voxe represents a valuable and promising solution to systematically integrate patient-related outcome (PRO) data into complex and dynamic clinical health care settings.

**International Registered Report Identifier (IRRID):**

RR2-10.1136/bmjopen-2021-053119

## Introduction

### Patient-Reported Outcome Measures

Patient-reported outcome measures (PROMs) are gaining significant momentum in clinical practice and research to foster a patient-centered approach to health care delivery [1-4]. PROMs are questionnaires used to collect subjective information directly from the patient regarding their health-related quality of life (HRQoL), functional status, and symptoms [5,6]. By directing the foci of clinical encounters, PROMs can facilitate early detection and monitoring of patient symptoms, empower patients to actively participate in their care, enhance health care providers’ (HCPs) understanding of patient needs, and influence joint discussions with patients about health outcome priorities [3,4]. In documenting patient-reported progress, PROMs can guide treatment decisions, positively influence patient outcomes [7], and encourage measurement-based care practices [8].

Despite the proposed value of PROMs, low PROM adoption rates have been attributed to factors related to the completion of paper-based PROMs, including limited time and resources among clinicians and low response rates from patients [4]. Digital electronic PROMs (ePROMS) have been designed to overcome cited barriers and to improve PROM data quality and completion time [1,9]. Many benefits have been documented such as greater patient preference and acceptability, higher data quality and response rates, and reduced health care costs [1]. Currently, platforms used for ePROM collection (eg, REDCap [Research Electronic Data Capture]) primarily target the clinical care of adults [9-11], with few designed specifically for the clinical management of children’s physical, social, and emotional health [4,10,12-14]. The lack of child-friendly and age-appropriate ePROM platforms needs addressing as children as young as 5 have shown capacity to self-report on their HRQoL [15], and 8 years of age is the recommended age to administer self-reported measures to children [10]. In the context of limited pediatric ePROM platforms, and with considerations around regulatory data privacy and management guidelines, secure servers for data storage and timely, responsive administrative and technical support [16,17], the development of new evidence-based platforms that enhance eHealth solutions for pediatric care should be prioritized.

### User-Centered Design

User-centered design (UCD) is a popular design approach for developing eHealth innovations, including ePROM platforms [18]. When applying a UCD approach to optimize usability, compliance, and adoption of ePROM platforms, end users (ie, patients and HCPs) and key stakeholders (eg, decision- and policy-makers) are involved in the platform design processes [9,19,20]. Specifically, UCD outlines that (1) designers should understand end users and user-specific tasks, and (2) design processes are iterative to involve multiple cycles of design, testing, and redesign [21]. With attention to these parameters, using a UCD approach to eHealth platform development can create technologies that are meaningful, manageable, and sustainable for their user and organizational health care systems, potentially impacting the implementation success of eHealth solutions [22,23].

### Voxe

Voxe is a pediatric, user-centered, and custom-built ePROM platform that is a progressive web application designed to integrate PROMs into the delivery of clinical care for pediatric patients with chronic health conditions [24]. Despite the profound and multidimensional impact of chronic disease on children’s HRQoL, objective outcome metrics (eg, morbidity and mortality) alone are frequently used to determine the success of clinical interventions and care [25,26]. Given the paucity of child-oriented ePROM platforms in health care [27,28], Voxe represents a novel and child-friendly ePROM platform to facilitate the systematic integration of children’s subjective evaluations regarding their physical, social, and emotional well-being in the delivery of care [24]. The advantages to having a custom-built progressive web application are (1) the ability to tailor the user experience specifically for pediatric patients, (2) to seamlessly integrate Voxe into the clinical workflow to reduce the barrier to ePROM completion, and (3) to ensure that Voxe is compatible with various electronic devices (eg, mobile phones, tablets, and computers). Additional distinguishing features include Voxe’s capacity to incorporate any PROM, its capability to be created in different languages and its potential to integrate with any electronic medical record (EMR). Notably, for the purpose of Phase 5, Voxe was integrated with the EMR Epic.

### Objectives

**Figure 1:**
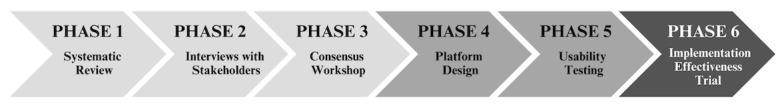
Overview of key phases involved in Voxe’s development.

Building on previously completed phases of Voxe’s development (Figure 1) [25,29], this paper outlines Voxe’s user-centered design approach (Phase 4) involving feedback from end users at 2 pediatric hospitals in Canada and subsequent usability testing (Phase 5) specific to Voxe’s platform features. The discussion will highlight our user-centered approach as a strength in prioritizing end user needs prior to Voxe’s implementation within a pediatric health care setting.

## Methods

### Study Participants and Inclusion Criteria

Purposive sampling was used to recruit patients followed by The Hospital for Sick Children (SickKids) Transplant and Regenerative Medicine Centre (TRMC) or Hematology and Oncology program at the Children’s Hospital of Eastern Ontario (CHEO) across age, diagnosis, sex, gender, and ethnicity. Members of the patients’ interdisciplinary health care teams at SickKids and CHEO were also purposively recruited across professional disciplines, years of practice, sex, gender, and ethnicity.

Patients were eligible if they met the following criteria: (1) heart, kidney, liver, or lung transplant recipients who were a minimum of 3 months posttransplant (SickKids) or followed by the Hematology and Oncology program (CHEO); (2) between 8 and 17 years of age; and (3) able to speak and read in English. Patients with significant cognitive impairments, as determined by a health care team member, were not eligible to participate. Eligible HCPs included any member of the interdisciplinary teams within the Hematology and Oncology program (CHEO; Phase 4) or the TRMC (SickKids; Phases 4 and 5).

### Ethical Considerations

Ethical approval to conduct this study was obtained from the Institutional Research Ethics Board at SickKids (1000057043 for Phase 4 and 1000067700 for Phase 5). All participants provided informed consent prior to their involvement in this study, and interview transcripts were deidentified. All participants received a $20 retail store gift card upon completion of study participation.

### Phase 4: Generation of the Voxe ePROM Platform

#### Overview

Previously completed phases of Voxe’s development process (Phases 1-3) [30,31] informed the design of preliminary Voxe wireframes. The PedsQL Generic Core Scales [32] were selected as the first ePROM to be designed within Voxe as it is considered the most widely used generic HRQoL pediatric PROM [33]. Voxe wireframes depicting the PedsQL Generic Core Scales adhere to the requirements noted in the e-Booklet for the Electronic Implementation of the PedsQL Generic Core Scales [34] and were reviewed personally by the lead original developer of the PedsQL.

Key stakeholders (eg, decision- and policy makers) were also consulted to identify Voxe users (ie, patients and HCPs) and the possible tasks end users would complete (ie, persona and task inventory development). Following the design of preliminary wireframes, a rapid and iterative testing methodology was used to evaluate and improve Voxe prior to its full development and launch [35]. Through a user-centered approach, one-on-one iterative testing sessions were conducted virtually with patients and HCPs by a member of the research team (SJP, SD) to elicit feedback on Voxe design features.

**Figure 2:**
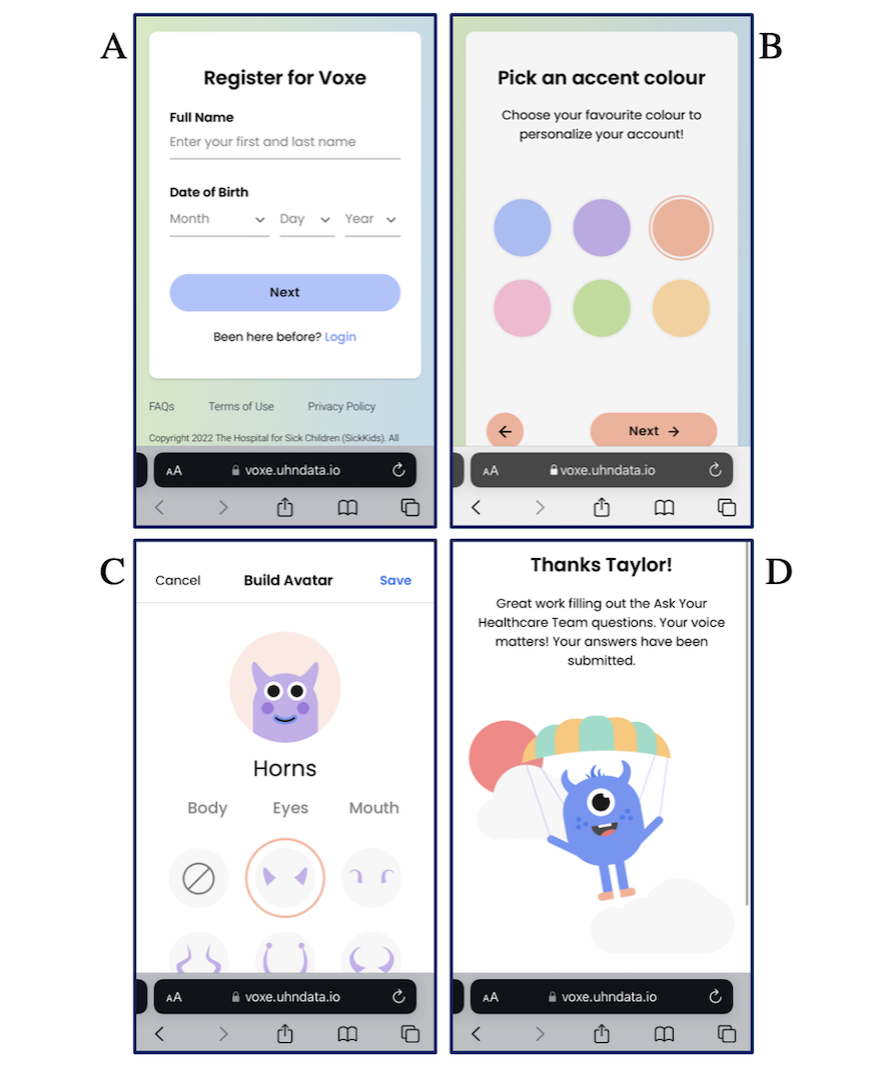
Examples of the Voxe Patient Registration (A), Personalization (B, C), and Ambassador GIF (D) Screens.

Patient features identified within Voxe for feedback included: (1) account personalization options, (2) text and email notifications, and (3) the display of Voxe ambassador GIFs. First, after registering for a Voxe account, patients have the option to personalize their account by selecting: (1) 1 of 6 accent colors which populate the header and buttons in the patient’s portal and (2) 1 of 6 prebuilt avatars or a custom-built avatar. Figure 2 presents the Voxe Patient Registration (A) and Personalization (B, C) screens. Second, Voxe allows patients to opt for text or email reminder notifications to complete the ePROMs on their preferred device (ie, mobile phone, tablet, or computer). Notifications are delivered by Voxe at 7 and 3 days in advance of clinic appointments. Third, following registration on Voxe and completion of each ePROM, the Voxe ambassador is displayed as a GIF (ie, animated avatar) on the platform. Figure 2 illustrates a still image of one of the Voxe ambassador GIFs (D) on a screen.

HCP features-of-use identified within Voxe for feedback included (1) the integration of Voxe with the EMR, (2) system (Epic) notifications of patient Voxe completion, and (3) a presentation view in Voxe to visualize trends in ePROM results over time. Notably, Voxe’s presentation screen was designed for HCPs to show patients a high-level summary of their ePROM results through graphs specific to each domain or summary score, depending on the ePROM. Table 1 summarizes patient and HCP features in Voxe.

**Table 1:** Patient and HCP^a^ features in Voxe.

Patient features	HCP features
Account personalization: select accent color, select a prebuilt avatar, and custom-build an avatar	Voxe integration with the EMR^b^
Text or email reminder notifications to complete ePROMs^c^	System notifications of patient Voxe completion
Display of Voxe ambassador GIF (ie, animated avatar) following registration and ePROM completion	Presentation view in Voxe to visualize trends in ePROM results over time

^a^HCP: health care provider.

^b^EMR: electronic medical record.

^c^ePROM: electronic patient-reported outcome measure.

#### Data Collection

Both patient and HCP participants completed task-based activities pertaining to the Voxe platform. Examples of tasks patients completed include: “This is your first time using Voxe. What would you click on to begin creating your profile?”; “What would you do if you wanted to pick a different colour?”; “You are doing the survey and let's assume you wanted to go back to a previous question. What would you click?” HCPs completed tasks such as “Let's assume that you need to view the patient’s PedsQL results from May 24, 2019. What would you click to access their past PROM results?”; “Let's now pretend you want to compare the patient’s PedsQL results between January 2019-June 2019 only. Where would you click to do this?”; “Voxe has a patient-friendly presentation feature to show patients their results during clinic and invite better conversation. Click where you would go to show the patient their patient-friendly overview of their results.” During HCP task-based activities, International Organization for Standardization key performance indicators (KPIs) were tracked, as KPIs are deemed essential for evaluating the introduction of a novel technology, technique, or process [36,37]. Objective and subjective standards common in user experience design testing [38] were collected to measure (1) effectiveness—accuracy and completeness with which users achieve specific goals, displayed as a percentage of tasks successfully completed by users, and (2) efficiency—resources used in relation to results achieved, represented by the time it takes users to complete standard tasks successfully [39].

Following the completion of task-based activities, HCP participants completed the System Usability Scale (SUS), a 10-item Likert scale questionnaire to assess the KPI satisfaction [40,41]. The SUS is considered a reliable way to evaluate electronic platforms, in which a score of 68 is considered above average [40,41]. Following the completion of task-based activities, patient participants selected 5 words from a list of product reaction words outlined by the Microsoft Desirability Toolkit [42] (eg, creative, easy, and friendly) to describe their overall impression and experience with the Voxe platform.

Qualitative data were collected through semistructured interviews during which participants shared their likes and dislikes of the Voxe platform design and commented on the platform’s ease of use and elements of functionality. Interviews were audio-recorded, transcribed verbatim, and deidentified.

### Phase 5: Usability Testing of the Voxe ePROM Platform

#### Overview

To test the usability of the Voxe platform, the think-aloud technique was used in which participants verbalized their thoughts and feelings while interacting with Voxe to complete structured tasks [43,44]. The think-aloud technique is a well-known, formative usability testing approach to identify usability issues in the user interface designs of technologies such as ePROM platforms [45-47]. The think-aloud technique was integral to understanding the end user experience with Voxe and highlighting potential barriers to Voxe adoption that will inform its subsequent implementation [43,44].

#### Data Collection

Following the development of interfaces of the Voxe ePROM platform for patients and HCPs, one-on-one iterative testing sessions were conducted virtually by research team members (SJP, AD, MA, SD, and SO) with patient and HCP participants. The purpose of the testing sessions was to obtain patients’ and HCPs’ feedback on the appearance and functionalities of the Voxe platform prototype.

During the first 2 testing rounds, patient participants were asked to complete a core set of tasks on Voxe, which were presented to them in the form of scenarios that they may encounter while interacting with Voxe. Examples of tasks patients completed include: “This is your first time using Voxe. Please make an account, enter a phone number and log into your account.”; “How would you set your profile colour as yellow?”; “A few weeks have passed since your appointment at the hospital. During your appointment your nurse mentioned you can see a summary of your results to the PROMs you answered earlier on Voxe. How would you view your results from surveys you have completed?” The last 2 testing rounds were conducted to simulate “real-world” settings. An automated text message or email with an embedded hyperlink was sent to patients asking them to access Voxe remotely on a smartphone, tablet, or computer. Patients independently logged into Voxe using an anonymous username and password and navigated the platform to complete the ePROMs.

HCP participants accessed Voxe on a computer to complete a core set of tasks which simulate scenarios they may encounter while using Voxe in clinical practice. HCPs completed tasks such as “Click where you would go to view the patient’s PedsQL results”; “Let's assume that you need to view the patient’s PedsQL results from an earlier date. What would you click to access patient’s past PROM results?”; “You are now meeting with the patient in clinic and would like to show them a quick summary of their PedsQL results. Click where you would go to share an overview of their results.” Using think-aloud methodology, patient and HCP participants voiced out loud what they were looking at, thinking, doing, and feeling as they navigated the platform [43,48].

**Figure 3:**
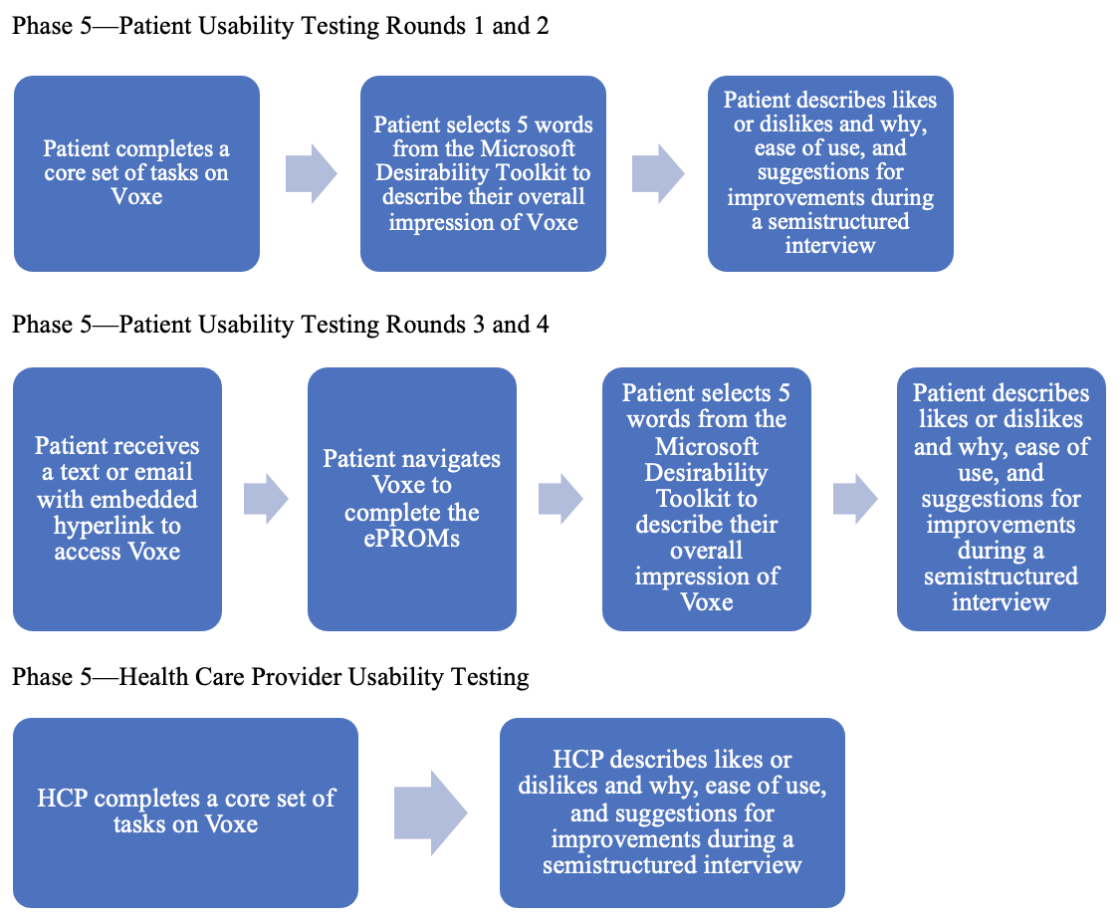
Overview of Phase 5 usability testing procedures for patients and health care providers. HCP: health care provider.

After completing task-based activities, patient participants selected 5 words from the Microsoft Desirability Toolkit [42] to describe their overall impression and experience with the Voxe platform. Qualitative data were collected through semistructured interviews to elicit information on what patient and HCP participants liked or disliked and why, the ease of use, elements of functionality in the context of typical practice workflow, and suggestions for improvements. Interviews were audio-recorded, transcribed verbatim, and deidentified. Rounds of iterative feedback testing were conducted with each participant population until Voxe was considered acceptable to participating end users with no further refinements identified [49-51]. Figure 3 presents an overview of usability testing procedures for patients and health care providers.

### Data Analysis

#### Phase 4: Quantitative Data

Quantitative data from the platform creation testing sessions included (1) objective and subjective International Organization for Standardization KPIs, and (2) HCPs’ scores on the SUS questionnaire. Descriptive statistics were calculated and summarized as appropriate. The quantitative data were triangulated with qualitative data to provide a comprehensive understanding of end users’ experience with Voxe. Further refinements were subsequently made to the Voxe platform design based on the triangulated data.

#### Phases 4 and 5: Qualitative Data

Research team members experienced in qualitative methods (SJP, AD, MA, SD, and SO) used content analysis to identify and organize meaningful patterns into codes across the data collected from qualitative interviews [52-54]. Codes were primarily developed deductively from key concepts in the interview guides [55], and categories were created to identify areas of similarity by collapsing codes with unifying features [56,57]. Categories were reviewed and refined until a consensus was reached among team members. In the context of the current study, saturation as an end point criterion for completing data collection was determined when Voxe was considered acceptable to end users as measured by no further requests for refinements. NVivo 12 Lumivero was used for qualitative data management [58].

## Results

### Phases 4 and 5 Patient Participant Results

#### Patient Participants

A total of 49 patients from SickKids and CHEO participated in iterative testing rounds of Voxe in Phases 4 (platform creation) and 5 (usability testing) between September 2020 and August 2022. Among these, 19 patient participants were boys, 28 were girls, and 2 were nonbinary. Two participants were 8 or 9 years of age, 16 were between 10 and 13 years of age, and 31 were between 14 and 17 years of age. Participants identified as Asian (n=2); Black, Afro-Caribbean, or African American (n=4); Hispanic, Latino, or Spanish (n=1); South Asian (n=9); South Asian and White or Caucasian (n=4); White or Caucasian (n=28); and Other—White and Vietnamese (self-reported) (n=1). At SickKids, 12 and 13 participants participated in Phases 4 and 5, respectively. Of those, 8 participants were involved in both Phases 4 and 5. At CHEO, 12 participants participated in Phases 4 and 5. Eight participants were involved in both Phases 4 and 5. Table 2 reports additional patient participant demographic information.

**Table 2:** Patient demographics.

Variable		Phase 4 SickKids	Phase 4 CHEO	Phase 5 SickKids	Phase 5 CHEO	Total
**Participant sex, n**						
	Female	7	7	7	7	28
	Male	5	5	6	5	21
**Participant gender, n**						
	Boy	4	5	5	5	19
	Girl	7	7	7	7	28
	Nonbinary	1	0	1	0	2
**Participant age in years, n**						
	8 or 9	0	0	1	1	2
	10-13	3	6	4	3	16
	14-17	9	6	8	8	31
**Transplant type (SickKids only), n**						
	Kidney	4	—^a^	4	—	8
	Liver	4	—	4	—	8
	Lung	1	—	2	—	3
	Heart	3	—	3	—	6
**Time since transplant in years (SickKids only)/diagnosis in years (CHEO^b^ only), n**						
	<6	5	4	4	6	19
	6-10	0	3	3	1	7
	11-15	7	3	4	3	17
	>15	0	1	2	2	5
	12 and <1	0	1	0	0	1
**Diagnosis (CHEO only), n**						
	Oncological	—	3	—	3	6
	Bleeding disorder	—	6	—	4	10
	Red cell disorder	—	1	—	3	4
	Thrombotic disorder	—	2	—	2	4
**Ethnicity, n**						
	Asian	1	0	1	0	2
	Black, Afro-Caribbean or African American	0	2	0	2	4
	Hispanic, Latino, or Spanish	0	0	1	0	1
	South Asian	3	1	3	2	9
	South Asian and White or Caucasian	1	1	1	1	4
	White or Caucasian	7	7	7	7	28
	Other—White and Vietnamese (self-reported)	0	1	0	0	1

^a^Not applicable.

^b^CHEO: Children’s Hospital of Eastern Ontario.

#### Patient Participant Experience

##### Increased Motivation Through Account Personalization Options

Several patients shared that the variety in options for customizing their personal Voxe account was an attractive feature of the Voxe platform to enhance their motivation to complete the ePROMs. Patients also appreciated that their personalization selections could be amended at any time. The personalization component was described as unique to Voxe, as one participant stated: “My favorite part was building the avatar… Because I think it’s what makes it different from other apps…it kind of just felt like you’re creating an avatar for a video game, which is pretty fun… [it] like makes doing something for the hospital actually fun” (Usability Testing SickKids #5).

##### ePROM Completion Facilitated Through Text and Email Notifications

Patients stated a collective preference for Voxe’s text notifications on the basis of checking text notifications more frequently than email notifications and being more familiar with texts. Patients also emphasized the convenience of notification alerts appearing on their mobile phone screens rather than being embedded in an email, as one patient stated that they might be “more inclined to miss it [the notification to complete their ePROMs in Voxe] if it was on… email…” (Design Testing CHEO #2). Despite an overarching preference for mobile phone notifications, select patients described the value of offering children a choice of email or text notifications, depending on personal preference. Overall, patients reported that the text and email notification feature of the Voxe platform helped facilitate their ability to complete the ePROMs.

##### Positive Reinforcement Fostered Through the Voxe Ambassador GIFs

Nearly all patients expressed that the ambassador’s presence in the platform reinforced a unique and child-centered sense of delight, engagement, and accomplishment regarding completing ePROMs, as noted by one patient: “If you’re feeling down and you saw those animations... it’ll probably cheer you up” (Design Testing CHEO #1). Several participants also noted that completing ePROMs in Voxe represents a psychosocial intervention in and of itself to uplift their mood, as one patient shared: “You did what you had to do, and you did it perfectly… it makes you feel better” (Usability Testing CHEO #4).

### Phases 4 and 5 Health Care Provider Participant Results

#### Health Care Provider Participants

A total of 38 HCPs from SickKids and CHEO participated in iterative testing rounds of Voxe (Phases 4 and 5) between April 2020 and January 2023. Of these, 6 HCPs were men and 32 were women. Eleven participants had worked in the SickKids TRMC or at CHEO between 6 months and 5 years, 9 participants had worked there between 6 years and 10 years, 4 participants had worked there between 11 and 15 years, 9 had worked there between 16 and 20 years, and 5 had worked there over 20 years. HCP participants identified as Asian (n=3); Asian and White or Caucasian (n=1); Black, Afro-Caribbean or African American (n=3); South Asian (n=1); White or Caucasian (n=29); and Other—Greek (self-reported) (n=1). Table 3 reports additional HCP participant demographic information. At SickKids, 3 participants were involved in both Phases 4 and 5.

After completion of design testing, 94% (30/32) of HCPs described Voxe as being user-friendly, and 88% (28/32) felt that most people would learn to use Voxe quickly. Task success or effectiveness increased by 11.25% from Round 1 to Round 4. Task completion rate decreased by 7.34 seconds over the 4 rounds of testing. SUS or satisfaction increased from a B (75.94) to an A (83.75) between Round 1 and 4. Table 4 comprises task success, task completion, and system usability score metrics.

**Table 3:** HCP^a^ demographics.

Variable		Phase 4 SickKids	Phase 4 CHEO^b^	Phase 5 SickKids	Total
**Participant sex, n**					
	Female	13	14	5	32
	Male	3	2	1	6
**Participant gender, n**					
	Man	3	2	1	6
	Woman	13	14	5	32
**Clinician type, n**					
	Dietician	1	0	0	1
	Nurse	2	6	2	10
	Nurse practitioner	3	1	1	5
	Occupational therapist	1	0	0	1
	Physician	4	5	2	11
	Physician assistant	0	2	0	2
	Physiotherapist	1	0	0	1
	Psychologist	1	1	0	2
	Social worker	2	1	1	4
	Other—information coordinator	1	0	0	1
**Transplant program or area of work (SickKids only; participants reported all areas of work; total participants: 16 in Phase 4 and 6 in Phase 5), n**					
	GIFT^c^	1	—^d^	0	1
	Heart	5	—	0	5
	Kidney	6	—	6	12
	Liver	8	—	0	8
	Lung	5	—	0	5
	Small bowel	1	—	0	1
	Other—intestine	1	—	0	1
**Department or area of work (CHEO; total: 16 participants), n**					
	Department of Pediatrics	—	2	—	2
	Hematology	—	2	—	2
	Hematology and Oncology	—	7	—	7
	MDU^e^	—	2	—	2
	Oncology	—	3	—	3
**Number of years working in the SickKids TRMC^f^ (SickKids only)/at CHEO (CHEO only)**					
	6 months to 5 years	2	7	2	11
	6 years to 10 years	4	4	1	9
	11 years to 15 years	4	0	0	4
	16 years to 20 years	3	3	3	9
	More than 20 years	3	2	0	5
**Ethnicity, n**					
	Asian	2	0	1	3
	Asian and White or Caucasian	0	1	0	1
	Black, Afro-Caribbean or African American	1	1	1	3
	South Asian	1	0	0	1
	White or Caucasian	12	13	4	29
	Other—Greek (self-reported)	0	1	0	1

^a^HCP: health care provider.

^b^CHEO: Children’s Hospital of Eastern Ontario.

^c^GIFT: Group for Improvement of Intestinal Function and Treatment

^d^Not applicable.

^e^MDU: medical day unit.

^f^TRMC: Transplant and Regenerative Centre.

**Table 4:** Task success, task completion, and system usability score metrics (Phase 4).

Metric	HCP^a^ Round 1	HCP Round 2	HCP Round 3	HCP Round 4
Task success (%), mean (SD)	74.17 (12.57)	75.00 (14.32)	79.17 (6.30)	85.42 (11.57)
Task completion (seconds), mean (SD)	23.15 (9.34)	17.05 (7.40)	15.77 (5.20)	15.81 (5.58)
System Usability Score (grade), mean (SD)	75.94 (B); (7.78)	71.88 (C+); (8.81)	73.13 (B-); (12.87)	83.75 (A); (11.73)

^a^HCP: health care provider.

#### Health Care Provider Participant Perspective

##### Integrating Voxe With the Electronic Medical Record Is Important

During design testing sessions, HCPs noted the importance of integrating Voxe with the EMR (eg, Epic) as opposed to a standalone ePROM platform to allow HCPs to access PRO data using the existing EMR portal. HCPs associated the value of Voxe’s capacity for immediate data transfer between Voxe and the hospital EMR with reducing technological fatigue and optimizing clinical workloads, particularly specific to documentation procedures. For example, 1 HCP described: “I think it would really be very useful if you know that that link could happen between Voxe [and Epic] especially if we’re meant to write a plan and to basically get integrated and pull into the note… I think it has to be as seamless as possible” (Design Testing SickKids #5).

##### System Notifications of Voxe Completion Are Helpful

HCPs discussed the importance of system notifications in EMRs to signify when a patient completes their ePROMs within Voxe. According to HCPs, EMR system notifications would serve as a helpful reminder for HCPs to regularly check patients’ ePROM data in the EMR. Respective to notification placement within EMRs, HCPs preferred that the notification be viewed in a prominent area (eg, centered on the page) or in the form of an “In Basket” notification within Epic (“In Basket” is the communication hub in Epic). Several HCPs stated that an “In Basket” notification offered an added advantage of separating PRO data from other clinical data to ensure that ePROMs are reviewed by HCPs. This finding was captured by an HCP, who stated: “…the In Basket… it’s a little bit separate from like, say [clinical] results… I feel like if it was mixed in… that could easily get… missed” (Usability Testing SickKids #6).

##### Using the Presentation View to See Trends Within Results Over Time Is Valuable

HCPs emphasized the value of this feature of Voxe. In particular, HCPs commented on the presentation screen’s simplicity and ease of use to identify pertinent topics or issues that may require HCPs’ heightened attention during clinical encounters. HCPs also described that the presentation screen’s capacity to visualize ePROM results over different time points could represent a tool to facilitate conversations with patients and family members on issues related to HRQoL, including medication management and treatment adherence. For example, an HCP noted: “…if I can see consistently that their pain has affected their physical functioning… that’s a tool that I can use to say, ‘Well, look how you’re rating this, and you still don’t want to go on your medication?’… having a longer view is going to be even more powerful in our education and trying to help get buy-in with our plans” (Design Testing CHEO #8).

## Discussion

### Principal Findings

This paper provides an overview of a UCD approach and usability testing phases of a novel evidence-based pediatric ePROM platform prototype named Voxe. Patients particularly appreciated Voxe’s child-friendly options for personalized profiles (eg, color and avatar selection) and the inclusion of Voxe ambassador GIFs (ie, animated avatars) in the platform. HCPs’ SUS scores reflected high satisfaction rates with the Voxe platform prototype. Design testing sessions with HCPs also highlighted Voxe’s ease of use and unique capacity for integration into the hospital EMR as valuable for streamlining clinical documentation and evaluation processes. Patients’ and HCPs’ high satisfaction with the Voxe prototype highlight the importance of being responsive to user suggestions from the inception of eHealth platform developments to ensure their efficient and effective design [20,59].

### Comparison With Prior Work

Patients’ feedback on the Voxe prototype highlights the need to integrate developmentally responsive design considerations (eg, choice of color and avatars) in pediatric ePROM platforms to foster children’s sense of engagement and motivation to complete ePROMs. While children and adolescents may be particularly amenable to using eHealth platforms due to their familiarity with technologies such as the internet and mobile phones, eHealth solutions should still respond to children’s rapidly shifting development and associated ideas about the novelty or innovative nature of technology [60]. Insufficient consideration of the needs of the intended users in the development of eHealth platforms risks eHealth tools that are not able to fully accomplish their objectives [61], particularly if technologies do not align with end users' daily lives, habits, or rituals [22]. Patients’ insights on Voxe’s child-friendly characteristics contribute to a limited body of research regarding how to tailor ePROM platforms to the preferences of pediatric patients to optimize ePROM adoption and implementation [14,62,63].

The UCD approach that guided Voxe’s development responds to existing calls for user-centered approaches to pediatric eHealth solution developments [64,65]. UCD is recognized as an optimal design approach for creating eHealth platforms [18,66,67] to help overcome eHealth implementation barriers, such as minimal clinical use of eHealth tools and low adoption rates in health care practices [68]. As eHealth technologies are often developed with a marginal level of engagement from end -users [68], Voxe’s development phases offer practical steps for facilitating the inclusion of end users’ perspectives in creating eHealth platform solutions specific to pediatric health care.

One challenge in pediatric health care practice is that most oral and written communications “with” children occur between adults [69-71]. Voxe’s usability testing sessions highlighted Voxe’s capacity for motivating pediatric patients to complete ePROMs and the potential to engage them in discussions concerning their care and treatment. For example, Voxe’s development directly responded to what children considered most meaningful to them (eg, the incorporation of account personalization and Voxe ambassador GIFs), which may differ from adult-informed ideas about children’s needs and preferences. Voxe also offers children time to reflect on pertinent issues they wish to emphasize in upcoming clinic appointments, and HCPs can review and consider patients’ responses before clinical visits. Participants highlighted that Voxe’s reminder notification system and presentation screen, which display the patient’s summarized ePROM results, could be useful tools to facilitate child-provider communication regarding PRO data. The design and functionalities of the Voxe prototype responded to patient needs within the context of clinic visits, encouraging in-advance completion and purposively directing the foci of clinical encounters to patient priorities, offering the potential to improve satisfaction with health care [72-74].

The integration of ePROM data into EMRs presented another contextual variable driving ePROM platform development to address end user needs. Recent estimates suggest that most ePROM platforms are designed as stand-alone platforms, and only 60% (6/10) of ePROM systems offer compatibility for linking with EMRs [75]. In the context of limited pediatric ePROMs, Voxe is one of only a few pediatric-focused [4,76] or combined (ie, children and adults) [10] ePROM platforms to offer the capacity for front-end integration with EMRs. Similar to other cited benefits of ePROM platforms [6,72,74], Voxe can facilitate the integration of PRO data in patient EMRs in a manner that mirrors clinicians’ existing workflows relative to documentation and assessment practices. During Voxe’s usability evaluations, HCPs emphasized the value of Voxe’s capacity for seamless integration into the EMR, which facilitated viewing and sharing mock PRO data in real time, and has the potential to offer time-saving benefits and remove potential burdens associated with logging into a stand-alone platform to review PRO data [74]. Overall, HCPs described how Voxe could help address key barriers pertaining to ePROM uptake in pediatric clinical practices, such as inconsistencies in compatibilities with EMRs, which has been cited among other associated challenges with PRO data management [6].

### The Future Implementation of Voxe

The UCD approach and usability testing of Voxe outlined in this paper will inform the full operationalization and implementation of Voxe in pediatric health care settings. Future implementation initiatives will include (1) the delivery of HCP orientation sessions to familiarize HCPs with Voxe and (2) the evaluation (Phase 6) of the Voxe ePROM platform using a hybrid implementation-effectiveness design. Presently, the implementation of ePROMs in health care settings remains sparse and inconsistent [6], and transparent reporting on the use of implementation strategies to guide the future implementation of Voxe [77,78] will contribute to addressing this knowledge gap.

### Strengths and Limitations

Voxe was designed and evaluated by an interdisciplinary research team with expertise in pediatric health research, including UCD and mixed methodology. This approach elicited participant perspectives through qualitative methods, garnering insights from a diverse sample of end user participants (ie, patients and HCPs) relative to age and clinician type. Participants completed simple tasks aligned with using Voxe which provided feedback about the changes needed prior to implementation. Evaluating the use of Voxe in more complex, “real-world” situations and to interpret patient ePROM results and trends longitudinally is a future aim of our program of research. We acknowledge that participants represented a small sample recruited from the SickKids TRMC and the Hematology and Oncology program at CHEO which limited eligible chronic health conditions and may have implications on the generalizability of our findings. Future studies should include participation from other pediatric hospitals and clinical programs. Of note, the sample recruited included English-speaking participants only. The absence of the perspectives of non-English speaking individuals to inform the development of Voxe limits our transferability of findings within these populations.

### Conclusions

This paper outlines Voxe’s UCD approach and the usability testing of Voxe’s platform features as an ePROM platform designed for implementation into clinical care delivery for pediatric patients with chronic health conditions. As a patient-facing platform that can be integrated into EMRs, Voxe aligns with measurement-based care practices to foster quality patient-centered approaches to care. End users’ positive feedback and evaluation of the platform’s user-friendliness and efficiency suggest that Voxe represents a valuable and promising solution to systematically integrate PRO data in complex and dynamic clinical health care settings. Future collection of usage and outcome data will enable cost-benefit analyses to support the long-term integration of eHealth platforms in clinical services [79].

## Abbreviations

CHEO: Children’s Hospital of Eastern Ontario
ePROM: electronic patient-reported outcome measure
EMR: electronic medical record
HCP: health care provider
HRQoL: health-related quality of life
KPI: key performance indicator
PRO: patient-reported outcome
PROM: patient-reported outcome measure
REDCap: Research Electronic Data Capture
SUS: System Usability Scale
TRMC: Transplant and Regenerative Medicine Centre
UCD: user-centered design
